# Butyrylcholinesterase Levels on Admission Predict Severity and 12-Month Mortality in Hospitalized AIDS Patients

**DOI:** 10.1155/2018/5201652

**Published:** 2018-03-15

**Authors:** Lijun Xu, Biao Zhu, Ying Huang, Zongxing Yang, Jia Sun, Yan Xu, Jinlei Zheng, Sabine Kinloch, Michael T. Yin, Honglei Weng, Nanping Wu

**Affiliations:** ^1^The State Key Laboratory for Diagnosis and Treatment of Infectious Diseases, The First Affiliated Hospital, School of Medicine, Zhejiang University, Qingchun Rd, Hangzhou, China; ^2^Collaborative Innovation Center for Diagnosis and Treatment of Infectious Diseases, The First Affiliated Hospital, School of Medicine, Zhejiang University, Qingchun Rd, Hangzhou, China; ^3^Department of HIV/AIDS, Xixi Hospital, Hengbu Rd, Hangzhou, China; ^4^Zhejiang Provincial Center for Disease Control and Prevention, Xincheng Rd, Hangzhou, China; ^5^Department of HIV Medicine, Royal Free Hospital, Pond Street, London, UK; ^6^Division of Infectious Diseases, Columbia University Medical Center, New York, NY, USA; ^7^Department of Medicine II, Section Molecular Hepatology, Medical Faculty Mannheim, Heidelberg University, Mannheim, Germany

## Abstract

**Background:**

Butyrylcholinesterase (BChE) is synthesized mainly in the liver and an important marker in many infectious/inflammatory diseases, but its role in acquired immunodeficiency syndrome (AIDS) patients is not clear. We wished to ascertain if BChE level is associated with the progression/prognosis of AIDS patients.

**Methods:**

BChE levels (in U/L) were measured in 505 patients; <4500 was defined as “low” and ≥4500 as “normal.” Associations between BChE level and CD4 count, WHO stage, body mass index (BMI), C-reactive protein (CRP) level, and duration of hospitalization were assessed. Kaplan–Meier curves and Cox proportional hazards model were used to assess associations between low BChE levels and mortality, after adjustment for age, CD4 count, WHO stage, and laboratory parameters.

**Results:**

A total of 129 patients (25.5%) had a lower BChE level. BChE was closely associated with CD4 count, WHO stage, CRP level, and BMI (all *P* < 0.001). Eighty-four patients (16.6%) died in the first year of follow-up. One-year survival was 64.5 ± 4.5% for patients with low BChE and 87.6 ± 1.8% for those with normal BChE (log-rank, *P* < 0.001). After adjustment for sex, age, BMI, WHO stage, and CD4 count, as well as serum levels of hemoglobin, sodium, and albumin, the hazard ratio was 1.8 (95% confidence interval, 1.0–3.2) for patients with a low BChE compared with those with a normal BChE (*P* = 0.035).

**Conclusion:**

BChE level is associated with HIV/AIDS severity and is an independent risk factor for increased mortality in AIDS patients.

## 1. Introduction

The digestive system plays an important part in pathogenesis of infection by the human immunodeficiency virus (HIV) [1]. The intestinal mucosa and gut-associated lymph nodes can serve as “HIV reservoirs” [2, 3]. As the largest organ in the digestive system, the liver is associated with HIV infection. For example, dendritic cell-specific intercellular adhesion molecule-3-grabbing nonintegrin is expressed abundantly on liver sinusoidal endothelial cells and promotes HIV infection [4]. Furthermore, the liver can modulate several processes in HIV infection by regulating lymphocyte functions, especially those of liver-associated lymphocytes, which contributes to the pathogenesis of acquired immune deficiency syndrome (AIDS) [5].

On the other hand, HIV infection exerts direct and indirect effects on the liver. Studies have demonstrated that HIV can infect hepatocytes, Kupffer cells [6, 7], and hepatic stellate cells (HSCs) [8]. HIV infection of these three types of liver cells induces production of inflammatory cytokines, as well as increasing the risk of hepatic steatosis [9, 10]. As a result, circulating levels of several proteins synthesized in the liver (e.g., albumin, prealbumin, and transferrin) decrease in response to injury and inflammation in many critically ill patients, and attenuation of levels of such proteins is associated with an increased risk of mortality in AIDS patients [11].

Previously, we showed that 85% of hospitalized HIV/AIDS patients in China have advanced HIV disease and have nearly 25% mortality within the first year of highly active antiretroviral therapy (HAART), especially within the first 6 months of HAART [12]. Thus, identification of an easily obtainable biomarker that can predict short-term mortality would be an important advancement for care.

Butyrylcholinesterase (BChE) is an important enzyme synthesized in the liver. In China, serum BChE is recognized as a parameter of liver function included in nearly all the patients' routine chemical profile. The normal range of BChE is roughly from 4500 U/L to 15,000 U/L in adult Chinese population, and measurement of serum BChE is very cheap (≈1dollar). It is usually as a prognostic biomarker of liver diseases such as viral hepatitis, cirrhosis, hepatocellular carcinomas, and even liver failure [13–15]. BChE is also an important clinical marker in inflammation [16, 17], severe bacterial infection [18], and fungal infection [19]. Importantly, reduced BChE indicates severe systemic inflammation in critically ill patients [20]. In addition, the BChE level reflected the nutrition state of patients [21]. Interestingly, patients infected with HIV and either HBV or HCV showed a direct correlation between decreased BChE and reduced CD4 counts [22]. However, the relationship between BChE and AIDS is not clear. Since low BChE reflects poor immune status, increased inflammation, infections, and poor nutrition of patients, we hypothesize that BChE may be a potential biomarker for HIV/AIDS patients.

In the present study, BChE was employed to evaluate the progression and prognosis of AIDS patients and compared it against other commonly available biomarkers. We want to know if BChE level was associated with the progression/prognosis of AIDS patients.

## 2. Materials and Methods

### 2.1. Ethical Approval of the Study Protocol

The study protocol was in accordance with the 1975 Declaration of Helsinki and approved by the Ethics Committee of the First Affiliated Hospital, School of Medicine, Zhejiang University (Hangzhou, China). Written informed consent was obtained from all patients to participate in the study.

### 2.2. Study Cohort

**Figure 1:**
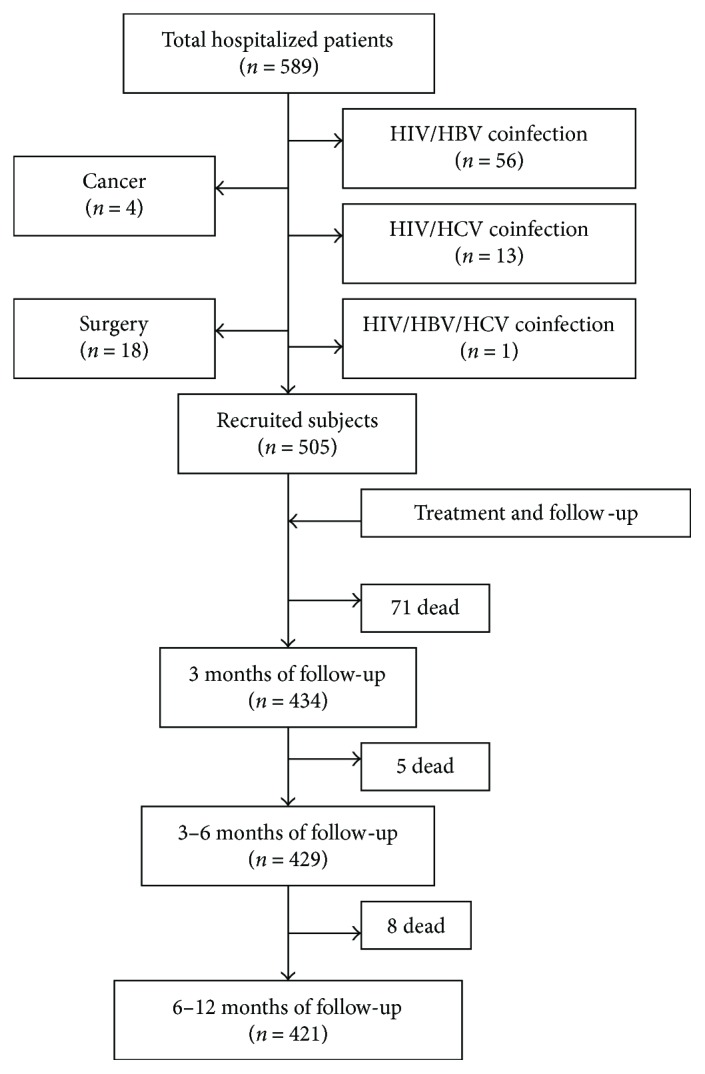
Study flowchart for patient selection.

Between April 2010 and March 2015, a total of 589 HIV/AIDS patients from the First Affiliated Hospital, School of Medicine, Zhejiang University, were eligible for enrollment in this prospective study. Among them, 84 were excluded for the following reasons: 56 had HIV/hepatitis B virus (HBV) infection, 13 had HIV/HCV infection, 1 patient had HIV/HBV/HCV infection, 4 patients had cancer, and 18 patients were about to undergo surgery. Thus, 505 patients formed the study cohort (Figure 1). Subjects underwent HAART if they had a CD4 cell count ≤ 350 cells/*μ*L or were WHO disease stage III or IV [23, 24]. The basic regimen was zidovudine (AZT) or tenofovir (TDF) plus lamivudine (3TC) combined with either nevirapine (NVP), efavirenz (EFV), or ritonavir-boosted lopinavir (LPV/r).

### 2.3. Laboratory Tests

Upon hospital admission, blood samples were drawn after a 12-hour fast. BChE was assayed with an Autobiochemical Analyzer (Beckman Coulter, Fullerton, CA, USA) using the choline thiobutyrate method. BChE ≥ 4500 U/L was defined as “normal BChE” and BChE < 4500 U/L as “low BChE.” Numbers of CD4+ and CD8+ T cells were measured using a Flow Cytometer (Becton Dickinson, Fullerton, NJ, USA) with fluorescein isothiocyanate-conjugated antihuman CD4, phycoerythrin-conjugated antihuman CD8, and phycoerythrin-Cy5-conjugated antihuman CD3 monoclonal antibodies (Becton Dickinson). HIV-1 RNA was assayed according to a standard protocol of the COBAS® AmpliPrep/TaqMan® 48 Analyzer (Roche, Basel, Switzerland). The lower limit of detection for HIV was 400 copies/mL.

### 2.4. Follow-Up and Collection of Clinical Data

Follow-up was undertaken at three-month intervals according to a method described previously [12, 25]. The time of follow-up was from the first day of hospital admission. Cases were followed up for 1 year.

### 2.5. Statistical Analyses

Continuous normal variables are the mean ± standard deviation. Categorical variables are the number of cases (percentage). CD4 count (cells/*μ*L) are expressed as medians (interquartile range, IQR). HIV-RNA levels (copies/mL) were log10-transformed into variables (log copies/mL) to meet the normality criteria for statistical analyses. Continuous variables were compared by one-way ANOVA or Student's *t*-test. Categorical variables were compared by *χ*2 analyses or Fisher's exact test. The effect of BChE on patient survival was analyzed by the Kaplan–Meier method and Cox proportional hazards model. “AIDS-related death” was defined as an “event.” Data for patients were censored at the date of the final visit (for those alive at the end of the follow-up period), date last known to be alive (for those with unknown vital signs), or the date of participants for whom the cause of death was not known to be AIDS-related. The clinical laboratory date collected were date only within the first three days of patients' admission. Data not available or beyond the first three days of patients' admission were defined as “missing data.” BChE (U/L) had categories of <4500 and ≥4500; age (years) of <30 and ≥30; WHO stage of I, II, III, and IV; CD4 count (cells/μL) of missing data, <50 and ≥50; HIV-RNA (copies/mL) of missing data , <10^5^ and ≥10^5^; hemoglobin (g/L) of missing data, <110 and ≥110; serum concentration of sodium (mmol/L) of missing data, <130 and ≥130; and albumin (g/L) of missing data, <35 and ≥35. These categories were included in the models as time-dependent covariates. These covariates were analyzed first in the univariate Cox model. Then, covariates with *P* < 0.2 in the univariate model were selected for the multivariate Cox proportional hazards model using the forward stepwise (likelihood ratio) method. The missing date was excluded for multivariate analysis. *P* < 0.05 (two-tailed) was considered significant. Data analyses were undertaken using SPSS v19.0 (IBM, Armonk, NY, USA) and Graphpad Prism version 5.0 (GraphPad Software, La Jolla, California, USA).

## 3. Results

### 3.1. Basic Characteristics of Patients

Five hundred and five patients formed the study cohort. Of these, 441 (87.3%) were male and 64 (12.7%) were female. Mean age of patients was 41.8 ± 13.8 years. Prevalence of patients with disease progression of WHO stage III or IV was 80.8%. Patients had advanced immunosuppression status with a median CD4 count of 76 (20–233) cells/*μ*L. Mean level of BChE of patients was 6073.8 ± 2280.8 U/L. Basic characteristics of patients are shown in Table 1.

### 3.2. BChE Was Closely Associated with HIV/AIDS Progression

**Figure 2:**
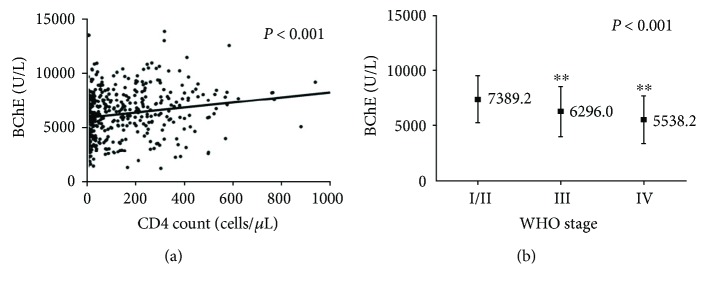
BChE is associated with HIV/AIDS progression. (a). BChE level is correlated with CD4 count (*P* < 0.001). (b). BChE level is negatively associated with HIV/AIDS stage (*P* < 0.001). ^∗∗^*P* < 0.01 using one-way ANOVA test.

In the present study, the CD4 count and WHO stage were used to indicate HIV/AIDS progression. Correlations among serum levels of BChE, CD4 count, and WHO stage were evaluated. A decrease in serum levels of BChE was positively correlated with CD4 depletion (*P* < 0.001) (Figure 2(a)). Serum levels of BChE and WHO stage of HIV/AIDS were also evaluated. The serum level of BChE (in U/L) was 7389.2 ± 2152.2 in patients with WHO stage I/II, 6296.0 ± 2261.4 for stage III, and 5538.2 ± 2138.2 for stage IV. Using ANOVA and LSD post hoc correction, we found that patients at WHO stage I/II had significantly higher BChE levels compared to the levels observed in patients at WHO stage III (*P* < 0.001) and WHO stage IV (*P* < 0.001). The serum level of BChe was negatively correlated with WHO stage (*P* < 0.001) (Figure 2(b)).

Additionally, HIV-RNA was tested in fifty-four patients in our study. No statistically significant correlations were found between BChE and HIV-RNA (*P* = 0.132).

### 3.3. BChE Was Significantly Correlated with Inflammation and Nutritional Status

**Figure 3:**
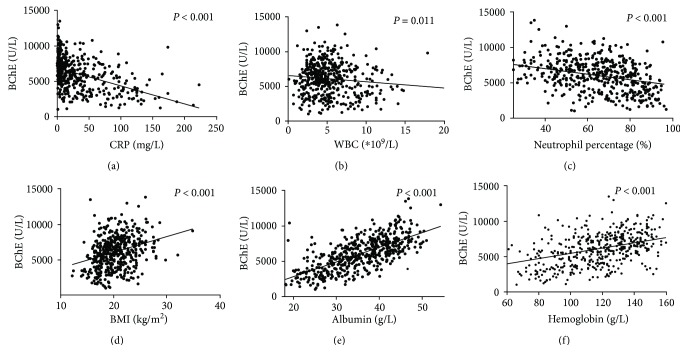
BChE, infection, and nutrition. BChE levels decrease significantly with increasing CRP levels, WBC, and neutrophil proportion (*P* < 0.001, *P* = 0.011, and *P* < 0.001, resp.) (a–c). BChE levels are positively associated with body mass index, albumin, and hemoglobin (all *P* < 0.001) (d–f).

White blood count (WBC), neutrophil proportion, and C-reactive protein (CRP) were used as indicators for inflammation/infection in patients. We assessed the relationships between serum levels of BChE and WBC and neutrophil proportion as well as CRP. Pearson correlation analyses suggested that a decrease in the serum level of BChE was negatively correlated with the CRP level (*P* < 0.001), WBC (*P* = 0.011), and neutrophil proportion (*P* < 0.001) (Figures 3(a)–3(c)).

We used the body mass index (BMI), serum albumin, and hemoglobin to evaluate the patients' nutrition status. The relationships between the serum level of BChE and BMI and serum albumin and hemoglobin were also studied. Correlation analyses suggested that the serum level of BChE was closely associated with the BMI (*P* < 0.001), serum albumin (*P* < 0.001), and hemoglobin (*P* < 0.001) **(**Figures 3(d)–3(f)).

### 3.4. Serum Level of BChE Is Associated with Treatment Outcome

**Figure 4:**
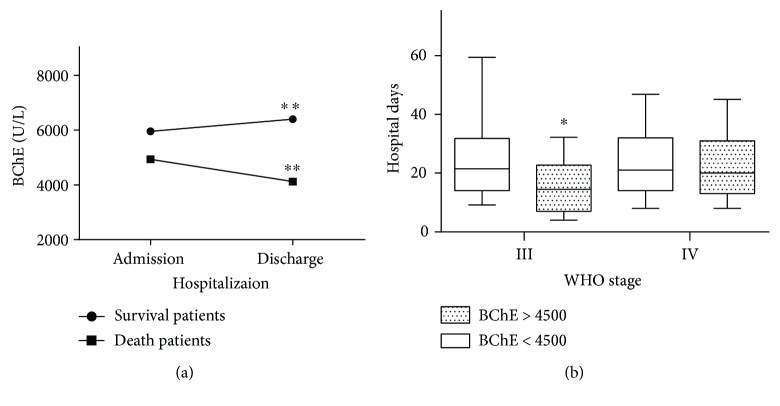
BChE level and hospitalization. (a) Mean BChE level increased from 5951.3 ± 2089.7 U/L to 6399.1 ± 1960.5 U/L (*P* < 0.001) in patients who survived during the hospitalization, whereas, mean BChE level decreased from 4932.7 ± 2118.3 U/L to 4122.9 ± 2009.1 U/L (*P* = 0.004) who died during hospitalization. (b) Patients with a low BChE have a longer duration of hospital stay than patients with a normal level of BChE at WHO stage 3 (*P* = 0.010), but this trend is not obvious among patients at WHO stage IV. ^∗^*P* < 0.05, ^∗∗^*P* < 0.01 using *t*-test.

To illustrate further the effects of serum levels of BChE upon hospitalization, the serum level of BChE of patients upon hospital admission and that upon hospital discharge was analyzed among surviving and deceased patients, respectively. Mean serum level of BChE level (in U/L) for surviving patients was 5951.3 ± 2089.7 upon hospital admission and 6399.1 ± 1960.5 upon hospital discharge, respectively (*P* < 0.001). In contrast, mean serum level of BChE level (in U/L) decreased from 4932.7 ± 2118.3 at hospital admission to 4122.9 ± 2009.1 upon transfer to the mortuary among deceased patients, respectively, in patients who died during the hospital admission (*P* = 0.004) (Figure 4(a)). Among surviving patients at WHO stage III, the duration of hospital stay (in days) was 14.5 (range, 7.9–22.8) in patients with a serum level of BChE (in U/L) ≥4500 and 21.5 (14.0–31.8) in patients with a serum level of BChE < 4500 (*P* = 0.010). Among surviving patients at WHO stage IV, the duration of hospital stay (in days) was 20 (range, 13–31) with a serum level of BChE (in U/L) ≥4500 and 21 (14–32) in patients with a serum level of <4500 (*P* = 0.597) (Figure 4(b)).

### 3.5. Serum Level of BChE Is an Independent Predictor of 1-Year Mortality among Hospitalized HIV/AIDS Patients

**Figure 5:**
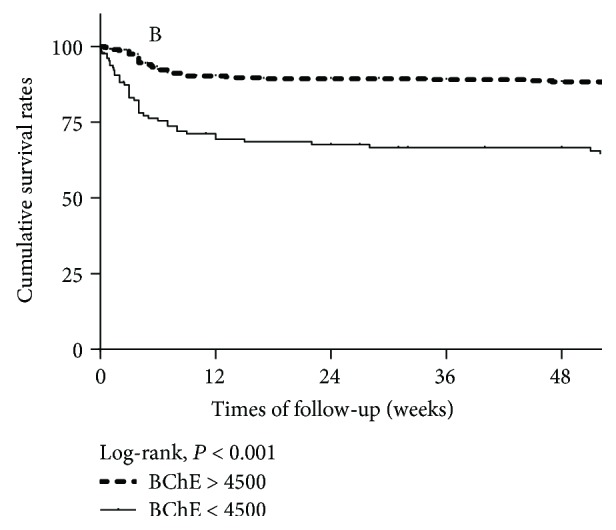
Kaplan–Meier survival curves according to BChE levels. The BChE level affects mainly 1-year mortality. One-year cumulative survival for patients with a low BChE is 64.5 ± 4.5% and 87.6 ± 1.8% for patients with a normal BChE level (log-rank, *P* < 0.001).

Of the 505 patients enrolled in our study, 84 (16.6%) patients had died at 1-year follow-up, including 42 (8.3%) patients with a low serum level of BChE and 42 (8.3%) patients with a normal serum level of BChE (*P* < 0.001). Seventy-one patients (14.1%) had died at 3-month follow-up. Five patients (1.0%) had died at 3–6-month follow-up, and 8 patients (1.6%) had died at 6–12-month follow-up. Kaplan–Meier analyses revealed that 1-year cumulative survivals were 64.5 ± 4.5% for patients with a serum level of BChE < 4500 U/mL and 87.6 ± 1.8% for those with a serum level of BChE > 4500 U/mL (log-rank test, *P* < 0.001) (Figure 5).

### 3.6. Serum Level of BChE Is a Predictor of Survival in Patients with HIV/AIDS

We stratified patients according to the following criteria: age (years; <30 and ≥30), BMI (kg/m^2^; missing data, <18 and ≥18), WHO stage (I, II, III, and IV), CD4 count (cells/*μ*L; missing data, <50 and ≥50), serum level of hemoglobin (g/L; missing data, <110 and ≥110), serum level of albumin (g/L; <35 and ≥35), serum concentration of sodium (mmol/L; missing data, <130 and ≥130), and serum level of BChE (U/L; <4500 and ≥4500). In the unadjusted model, our data suggested that the following factors contributed to mortality: age, BMI, CD4 count, and WHO stage, as well as serum concentrations of sodium, hemoglobin, albumin, and BChE. Hazard ratios (HRs) were 3.5 [95% confidence interval (CI), 2.3–5.3] for patients with a low serum level of BChE compared to those with a normal serum level of BChE. Next, we analyzed 383 patients without data missing. In the multivariate Cox proportional hazards model adjusted for age, CD4 count, and WHO stage, as well as serum levels of sodium, hemoglobin, and albumin, our data suggested that, compared with patients with a normal serum level of BChE, the HR was 1.8 (95% CI, 1.0–3.2) for patients with a low serum level of BChE (*P* = 0.035) (Table 2). These data suggested that the serum level of BChE was a predictor of survival in patients with HIV/AIDS. Patients with a low serum level of BChE had a higher HR for mortality than those with a normal serum level of BChE.

## 4. Discussion

Measurement of BChE is a very cheap (about one dollar) and routine item in liver function test in nearly all Chinese inpatients. However, the relationships between BChE and HIV infection remain unclear. In this prospective study of hospitalized patients with HIV/AIDS, we found that a low level of BChE at time of admission was associated with a higher risk of in-hospital death and 1-year mortality. Even after adjustment for sex, age, BMI, WHO stage, and CD4 count, as well as serum levels of hemoglobin, sodium, and albumin in the multivariate model, a low level of BChE was associated with a twofold higher risk of death. BChE may be a useful biomarker to predict clinical outcomes in patients with HIV/AIDS.

Cholinesterases include acetylcholinesterase and BChE. Acetylcholinesterase is present mainly at the ends of cholinergic nerves, cholinergic neurons, and red blood cells. BChE is an *α*-glycoprotein found mainly in the liver and used frequently as a parameter of protein synthesis in this organ [26]. BChE levels are decreased in disorders such as severe liver disease, poisoning with organophosphate compounds, cancer, and cachexia, whereas increased levels of BChE have been reported in obesity, diabetes mellitus, uremia, hyperthyroidism, and hyperlipidemia [26–28].

BChE plays an important role both in acute and chronic inflammatory diseases. Previous study indicated that two kinds of cholinergic status responders, enhancers and suppressors, were found in acute inflammatory phage. Enhancers showed increased BChE levels, complete WBC recovery, and improved cholinergic status modulations to plasma IL-6 levels in terms of acute infection or inflammation, but suppressors did not [29]. Likewise, BChE activities and levels in serum are associated with chronic low-grade and severe inflammatory diseases [16, 17, 20]. Interestingly, BChE is also detectable in human brain, participating in cholinergic status modulation, and nerve-macrophage interaction. Overall, BChE plays an important role in controlling extrasynaptic signalling of the cholinergic anti-inflammatory pathway, parasympathetic dysfunction, and inflammation-related disease [30, 31].

BChE is used as a marker to predict the prognosis of several disorders (inflammatory diseases, infection, malnutrition, malignancy, critical illness, liver disease, and metabolic diseases [32]), but the role of BChE in HIV/AIDS is incompletely understood. Here, we evaluated the association between the serum level of BChE and the prognosis of HIV/AIDS patients. Our data demonstrated that ≈30% of such patients had a reduced serum level of BChE.

Three complex factors predispose to a reduction in serum levels of BChE in AIDS patients. First, malnutrition and wasting syndrome are associated with protein-energy malnutrition (PEM) and accounted for a reduction in the serum level of BChE. Studies have demonstrated that abnormal absorption in the intestine is a common feature in HIV patients with or without diarrhea [1, 33]. Intestinal dysfunction and inadequate intake of nutrients are the main reasons for weight loss and wasting syndrome in HIV/AIDS patients [34]. Serum levels of BChE, total protein, and albumin are lower in malnourished children with marasmus than those measured in normal children; these values tend to increase after 3 weeks of nutritional rehabilitation, and a similar trend in serum levels of BChE has been observed in undernourished adults [35]. Nutritional status may be compromised in PEM and has important adverse effects upon outcome. Some authors have suggested that serum levels of BChE are nutritional and prognostic markers [21]. In our study, most patients (≈80%) were at WHO stage III/IV, so most subjects were underweight or had wasting syndrome. Patients at the latter stages of critical illness lose their appetite, resulting in inadequate intake of nutrients. Also, we found that serum level of BChE was closely associated with the BMI, albumin, and hemoglobin, suggesting that BChE is a potential marker of nutritional status. A lower serum level of BChE reflects the decreased nutrition of patients, which is a predictor of a bad outcome.

Second, infection and inflammation can affect serum levels of BChE. Chronic inflammation plays an important part in the pathogenesis of untreated HIV infection [36]. Such inflammation can result in dysfunction of hepatic protein synthesis, leading to reduction of BChE production [16, 20]. Systemic infection also contributes to BChE attenuation; BChE levels are significantly lower in those with bacteremia and could be useful for early detection of sepsis [18]. Low levels of BChE have also been noted in deep fungal infections, possibly due to enrichment of blood flow through the liver or invasion of fungi (e.g., *Candida albicans*, *Candida tropicalis*) into the liver from the gut by penetration through degenerated barriers of gastrointestinal mucosa [37]. Also, HIV-mediated destruction of the gut mucosa leads to microbial translocation, which induces persistent local and systemic inflammation, thereby promoting HIV/AIDS progression [36]. Translocated microbial products also pass through the liver, contributing to hepatic damage and impaired synthesis of proteins [36]. Earlier studies have suggested two types of cholinergic status responders, named the enhancers and suppressors. In the settings of acute infection or inflammation, the former refers to elevated level of BChE and improved WBC status as well as ameliorated cholinergic status modulations towards level of plasma IL-6, while the latter didn't show any of the forementioned status. In our study cohort, most patients were immunosuppressed and suffering from severe opportunistic bacterial/fungal infections. Critical illness or infection promotes an inflammatory response that has a rapid, catabolic effect on hepatic protein synthesis. Serum levels of inflammatory cytokines such as interleukin-6, interferon-gamma, and activity of BChE well as BChE activity were not measured in the present study, but we revealed that BChE levels were negatively associated with CRP levels, WBC, and of neutrophils proportion, demonstrating that infection contributes to lower levels of BChE.

Third, HIV infection itself may directly or indirectly affect liver function [38]. HIV-RNA has been detected in sinusoidal cells and hepatocytes in vivo [6]. A high level of HIV-RNA is an independent factor associated with increased levels of alanine aminotransferase in HIV-only-infected patients [39], suggesting that HIV facilitates liver dysfunction. HIV can infect hepatocytes [6], Kupffer cells [7], and HSCs [8, 40]. In our study, HIV-RNA levels were measured only in 54 patients. However, no obvious correlations were found between levels of HIV-RNA and BChE. Studies have suggested that HIV infection can damage liver function by inducing hepatocyte apoptosis [41], thereby impairing the ability of Kupffer cells to clear the products of microbial translocation [7] and promoting HSC production of proinflammatory cytokines [40]. In HIV patients, a systemic increase in lipopolysaccharide levels caused by microbial translocation results in chronic immune activation and contributes to impairment of protein synthesis [42] (including BChE synthesis). Thus, a direct relationship between HIV-RNA and BChE might be hidden by the three factors mentioned above.

Importantly, our research suggests that BChE might be associated with treatment outcomes. Serum levels of BChE increased from hospital admission to hospital discharge among surviving patients. However, serum levels of BChE decreased in patients who died during the hospitalization. These data suggest that effective treatments (including anti-infection agents and nutritional treatment) probably promote BChE synthesis in the liver. BChE was also associated with duration of hospital stay. Median duration of hospital stay among patients with a normal level of BChE was lower than that among cases with a low level of BChE at WHO stage III but not at stage IV. This difference might be because patients at stage IV have more complications and infections (Table 1), which probably conceal the effect of BChE level on median duration of hospital stay.

The main limitation of our study was that first, we did not fully assess the relationship between levels of HIV-RNA and BChE. The HIV-RNA test is relatively expensive in China, and carrying out this test in all patients is difficult. Nevertheless, we and other researchers understand the potential value of HIV-RNA upon liver function. Another limitation was missing data, especially CD4 count which was missing in 14.1% of patients. This was because Chinese government implemented the National Free Antiretroviral Treatment Program (NFATP) in 2003, with a “**Four Free and One Care**” policy (providing free HIV test and CD4+ T cell tests; free antiretroviral treatment for AIDS patients; free drugs to prevent mother-to-child transmission; free education for AIDS orphans; and government care for AIDS patients who live in poverty) [43]. Therefore, some patients had free CD4+ T cell tests performed in local the CDC clinic every three months instead of at our hospital, and we were not able to have access to the CDC data. Other missing data were mainly because the data were not obtained within the first three day of patients' admission. However, our data showed that BChE levels were clearly associated with patients' mortality in the multivariate Cox proportional hazards model, indicating that BChE level is associated with HIV/AIDS severity and is an independent risk factor for increased mortality in Chinese HIV/AIDS patients.

## 5. Conclusions

Our research suggests that even though it is not a specific marker of AIDS, a lower level of BChE is an independent surrogate marker of disease progression in patients with HIV/AIDS patients. Patients with low serum levels of BChE have a longer duration of hospitalization and higher risk of mortality within 1 year. BChE is a commonly utilized biomarker in China and should be included as a laboratory assessment at time of hospitalization for HIV/AIDS patients. Patients with lower serum levels of BChE may benefit for intensive nutritional support, earlier initiation of antibiotic therapy, and strategies to reduce inflammation.

## Figures and Tables

**Table 1:** Patient basic characteristics (*n* = 505).

Parameter	Value
*Age (years) (mean ± SD)*	41.8 ± 13.8
*Sex [n(%)]*	
Male	441 (87.3%)
Female	64 (12.7%)
*CD4+ (cells/μL) [median (IRQ)]*	76 (20–233)
*WHO disease stage [n (%)]*	
I/II	97 (19.2)
III	119 (23.6)
Pulmonary tuberculosis	23 (4.6)
Bacterial pneumonia	18 (3.6)
Other diseases	—
IV	289 (57.2)
Pneumocystis pneumonia	106 (21.0)
Extrapulmonary tuberculosis	78 (15.4)
Cryptococcal meningitis	42 (8.3)
Severe bacterial pneumonia	38 (7.5)
Fungal infection in bloodstream	13 (2.6)
Recurrent septicaemia	10 (2.0)
Nontuberculosis mycobacteria	5 (1.0)
Lymphoma	17 (3.4)
Other diseases	—
*HIV-RNA (log_10_ copies/mL)* ^∗^	4.3 ± 1.4
*Serum BChE (U/L)*	6073.8 ± 2280.8
*Serum albumin (g/L)*	35.6 ± 7.5
*Body mass index*	20.4 ± 3.1
*Hemoglobin (g/L)*	116.8 ± 26.5
Serum sodium (mmol/L)	137.8 ± 4.6

BChE: butyrylcholinesterase; IQR: interquartile range; WHO: World Health Organization; ^∗^HIV-RNA was available in 54 patients.

**Table 2:** Risk factors for mortality of patients in univariate/multivariate Cox proportional hazards models.

Factor	Number (*n* = 505)	Deaths (*n* = 84)	Univariate			Multivariate		
			HR	95% CI	*P*	HR	95% CI	*P*
*Sex*					0.817			—
Male	441 (87.3)	74 (16.8)	1.0	0.9–1.1				
Female	64 (12.7)	10 (15.6)	1					
*Age (years)*					0.020			—
<30	123 (24.4)	12 (9.8)	1					
≥30	382 (75.6)	72 (18.8)	2.1	1.1–3.8				
*BMI (kg/m^2^)*								
Missing data	37 (7.3)	22 (59.5)						
<18	110 (21.8)	21 (19.1)	1.8	1.0–3.0	0.036			—
≥18	358 (70.9)	41 (11.5)	1					
*WHO stage*					<0.001			0.008
I/II/III	217 (43.0)	19 (8.8)	1.0			1.0		
IV	288 (57.0)	65 (22.6)	5.6	3.0–10.5		2.9	1.3–6.5	
*CD4 count (cells/μL)*					<0.001			0.007
Missing data	71 (14.1)	15 (21.1)						
<50	181 (35.8)	45 (24.9)	3.0	1.8–4.9		2.4	1.3-4.4	
≥50	253 (50.1)	24 (9.5)	1			1		
*Serum sodium (mmol/L)*					0.002			—
Missing data	42 (8.3)	5 (11.9)						
<130	28 (5.5)	10 (35.7)	1					
≥130	435 (86.1)	69 (15.9)	2.9	1.5–5.6				
*Hemoglobin (g/L)*					0.035			—
Missing data	33 (6.5)	4 (12.1)						
<110	166 (32.9)	37 (22.3)	1.6	1.0–2.5				
≥110	306 (60.6)	43 (11.7)	1					
*Serum albumin (g/L)*					<0.001			—
<35	237 (46.9)	60 (25.3)	3.2	2.0–5.2				
≥35	268 (53.1)	24 (9.0)	1.0					
*Serum BChE (U/L)*					<0.001			0.035
≥4500	376 (74.5)	42 (11.2)	1			1		
<4500	129 (25.5)	42 (32.6)	3.5	2.3–5.3		1.8	1.0–3.2	

BChE: butyrylcholinesterase; BMI: body mass index; HR: hazard ratio; WHO: World Health Organization.

## Additional Points

*Article's Main Point.* Butyrylcholinesterase (BChE) is associated with HIV/AIDS progression. Patients with low serum levels of BChE have a longer stay in hospital and higher risk of death. Assessment of serum levels of BChE provides a routine biochemical approach for predicting clinical outcome of Chinese HIV/AIDS patients.
